# Uncontrolled Diabetes Unmasking Diabetic Striatopathy: A Reversible Cause of Hyperkinetic Movement Disorder

**DOI:** 10.7759/cureus.90731

**Published:** 2025-08-22

**Authors:** Amir A Estil-las, Martena Grace, Chetna N Hirani, Tanisha Vora, Camelia Arsene

**Affiliations:** 1 Medical School, Ross University School of Medicine, St. Michael, BRB; 2 Internal Medicine, Trinity Health Oakland Hospital, Pontiac, USA; 3 Internal Medicine, St. Joseph Mercy Oakland Hospital, Pontiac, USA

**Keywords:** choreiform movement disorder, diabetes mellitus, diabetic ketoacidosis (dka), diabetic striatopathy, non-ketotic hyperglycemia hemichorea-hemiballismus syndrome

## Abstract

Diabetic striatopathy (DS) is a rare neurological complication of non-ketotic hyperglycemia. It is distinguished by the acute onset of hemichorea and hemiballismus, as well as distinct radiographic findings involving the contralateral basal ganglia. We present the case of a 61-year-old male patient with a history of poorly controlled diabetes mellitus who developed acute involuntary movements affecting the right upper and lower extremities. Computed tomography (CT) revealed a hyperdensity in the left basal ganglia, and T1 hyperintensity with mild diffusion-weighted imaging hyperintensities on MRI, consistent with DS. Patient history and laboratory analysis revealed diabetic medication non-compliance and a glycosylated hemoglobin (HbA1c) of 14% and a blood glucose level of 472 mg/dL, without evidence of ketosis. The patient was treated with insulin therapy, resulting in proper glycemic control and complete resolution of symptoms. This case underscores the importance of recognizing DS in patients with hyperglycemia and acute movement disorders, as timely diagnosis and metabolic correction can lead to full neurological recovery and prevent unnecessary interventions.

## Introduction

Non-ketotic hyperglycemia hemichorea-hemiballismus, or commonly known as diabetic striatopathy (DS), is a rare disease in which a non-ketotic hyperglycemic state causes involuntary hyperkinetic movements [1]. The precise pathophysiology remains unknown; however, some popular theories include decreased gamma-aminobutyric acid (GABA) levels in the basal ganglia secondary to the non-ketotic state and hyperviscosity secondary to hyperglycemia, disrupting the blood-brain barrier and causing metabolic damage, and possibly myelin destruction by gemistocytes [1-3]. DS has an estimated prevalence of 1 in 100,000 [4]. Risk factors associated with DS include Asian ethnicity, female sex, prolonged poorly controlled blood sugar, and retinopathy [5]. Treatment for DS includes strict glucose control and occasionally the use of antichorea medications like haloperidol, tetrabenzine, and various other anti-dopaminergic agents [6]. In this case, we present a gentleman with poorly controlled diabetes mellitus (DM), among other medical histories, with imaging evidence of DS.

## Case presentation

We present the case of a 61-year-old man with a prior medical history of poorly controlled DM, hypertension (HTN), coronary artery disease, and s/p right coronary artery stent placement, who presented to the emergency department due to a home glucometer reading of 550 mg/dL. On arrival, the patient experienced shortness of breath, dizziness, anxiety, altered mental status, and increased urinary frequency. Vital signs were within normal limits.

Upon further interview, the patient admitted to not taking his home diabetic medications: metformin and glipizide for over 40 days. He was spending time with his friend earlier in the day when the friend expressed concern because the patient was having rapid and hyperactive speech. Moreover, the patient reported worsening severity of involuntary right arm and right leg jerking for the previous three days. The jerking movements were described as sudden flinging and stretching of the right arm and right leg with an athetotic component of the right hand. 

The patient was admitted, and laboratory values shown in Tables 1, 2 demonstrated a blood glucose level of 472 mg/dL and a glycosylated hemoglobin (HbA1c) of 14%. Insulin therapy was initiated, and glucose levels were measured throughout the stay (Table 3). After initial stabilization, the patient underwent several imaging studies including CT angiography of the head and neck with and without contrast, CT head without contrast, CT angiography of the upper extremity with and without contrast, MRI brain with and without contrast and MRA of the neck with and without contrast. 

**Table 1:** Hospital Admission Labs Values in bold indicate abnormal results.

Admission Labs		Reference Ranges
Glucose	472 mg/dL	<140 mg/dL (non-fasting)
HbA1c	14%	<5.7%
Na	132 mmol/L	136-146 mmol/L
K	4.4 mmol/L	3.5-5.0 mmol/L
Cl	101 mmol/L	95-105 mmol/L
CO2	25 mEq/L	22-28 mEq/L
Anion Gap	6 mEq/L	2-12 mEq/L
BUN	18 mg/dL	7-18 mg/dL
Creatnine	1.57 mg/dL	0.6-1.2 mg/dL
eGFR	50 mL/min	>90mL/min
BUN/Cr	11.5	10-20
Calcium	8.3 mg/dL	8.4-10.2 mg/dL

**Table 2:** Hospital Admission Urine Analysis Values in bold indicate abnormal results.

Urine Analysis	
Color	Colorless
Clarity	Clear
Specific Gravity	1.028
pH	6
Leukocytes	Negative
Nitrite	Negative
Protein	Negative
Glucose	>=1000
Ketones	Negative
Urobilinogen	Normal
Bilirubin	Negative

**Table 3:** Blood Glucose Trend

Blood Glucose Trend	
Day/Time	Glucose level (mg/dL)
1 | 10:30 pm	472
2 | 12:35 am	370
2 | 4:04 am	244
2 | 7:27 am	229

**Figure 1:**
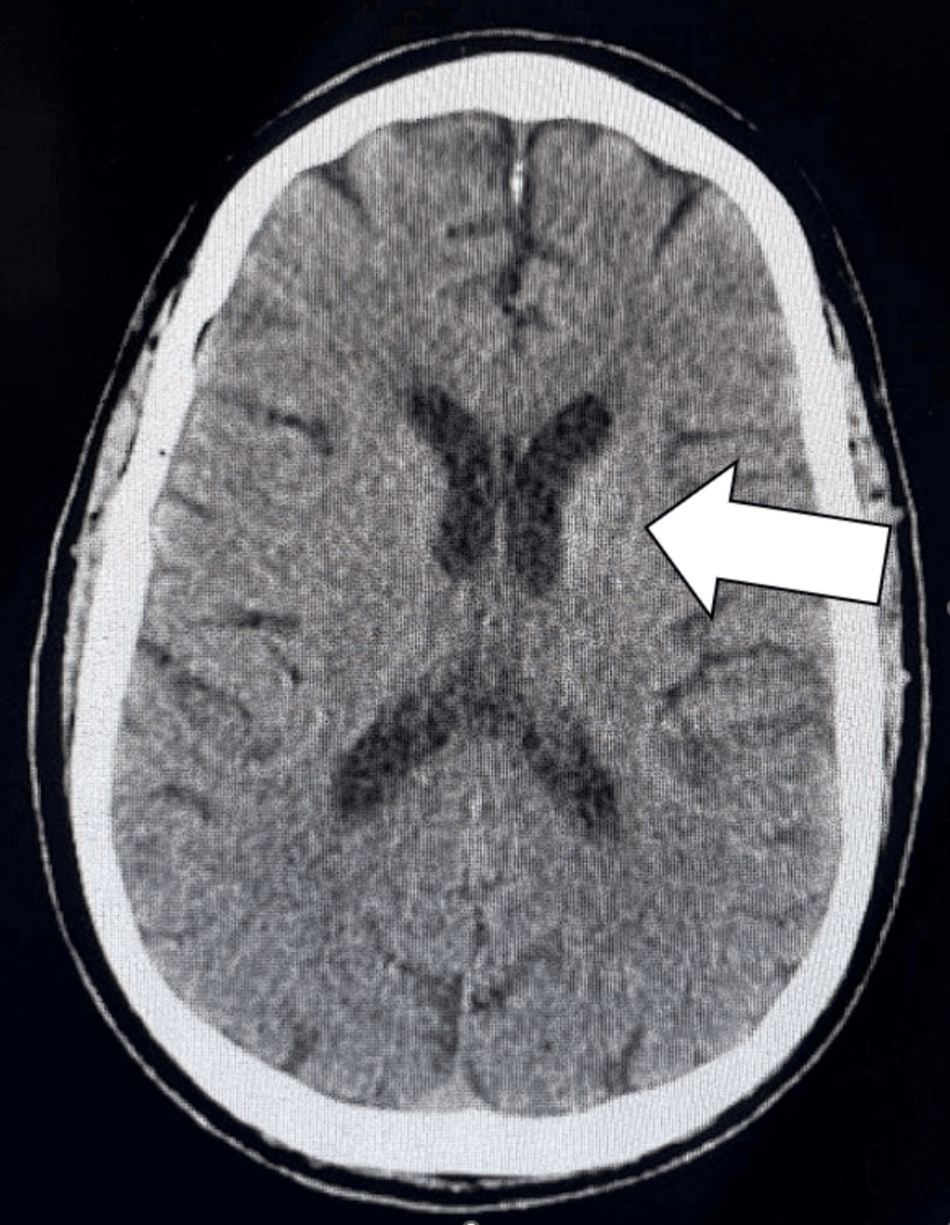
CT Scan of the Brain White Arrow: Prominent density on the left side of the patient's brain

**Figure 2:**
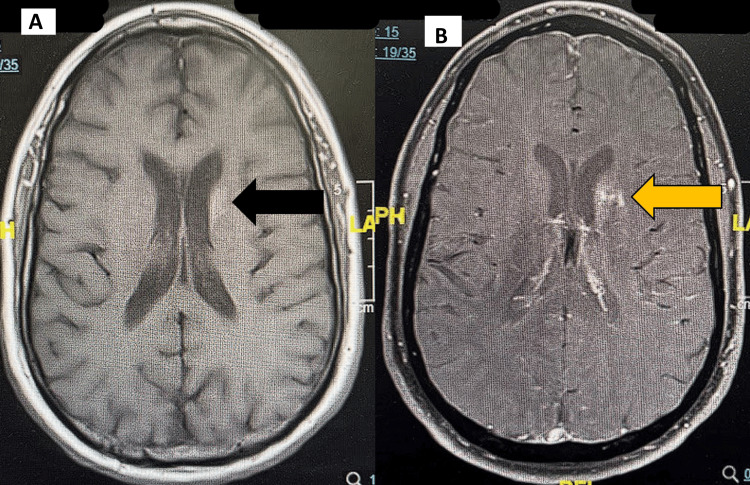
MRI T1 Sequence of the Brain (A): MRI T1 without contrast image of the brain. Black arrow pointing to hyperintensity in the left basal ganglia. (B): MRI T1 post-contrast enhancement image of the brain. Yellow arrow pointing to hyperintensity in the left basal ganglia.

**Figure 3:**
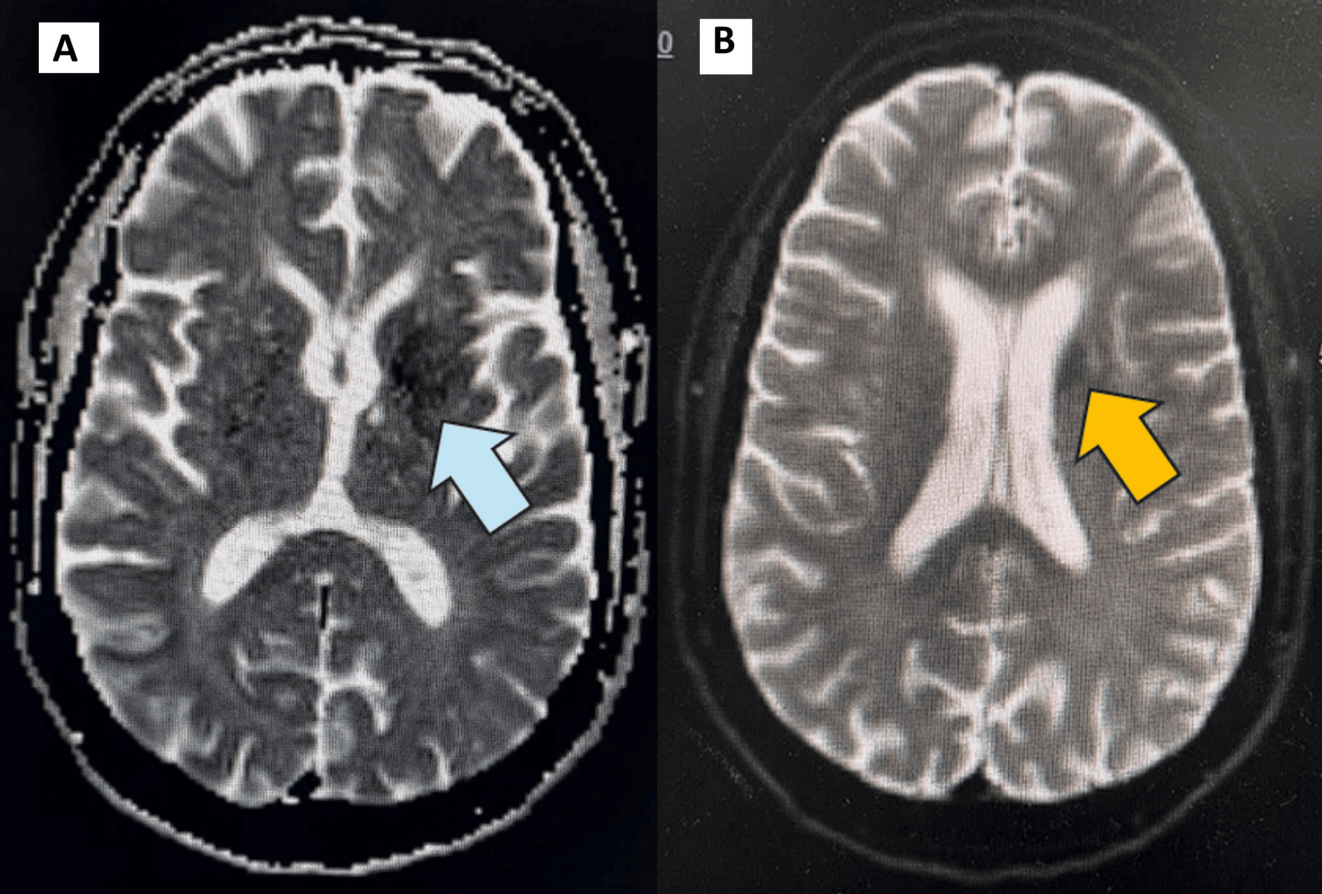
Brain MRI ADC and DWI Trace Sequences (A): Brain MRI ADC sequence. Blue arrow pointing at the left basal ganglia signal. (B): Brain MRI DWI trace sequence. Yellow arrow pointing at the left basal ganglia signal. ADC: Apparent diffusion coefficient; DWI: diffusion-weighted imaging

The pertinent findings identified on the imaging studies were on the CT head without contrast and MRI of the brain without contrast. CT showed an asymmetrical density of the basal ganglia more prominent on the left than on the right (Figure 1), suggesting non-ketotic hyperglycemia hemichorea-hemiballismus syndrome. MRI of the brain confirmed the diagnosis by identifying some T1 shortening abnormalities on the left basal ganglia (Figure 2), enhancement, and some diffusion-weighted imaging (DWI) hyperintensities (Figure 3).

After gradual correction of the patient’s blood glucose, the velocity of the involuntary movements decreased until an eventual cessation. The patient was discharged on metformin, glipizide, atorvastatin and sitaglipin, with follow-up appointments arranged with neurology, endocrinology, and primary care.

## Discussion

DS is a rare neurological complication of non-ketotic hyperglycemia, characterized by the acute presentation of involuntary hemichorea-hemiballismus, alongside pathomnemonic radiologic abnormalities in the contralateral basal ganglia. Hemichorea-hemiballismus is not exclusive to DS; in fact, several other pathologies may present similarly, some of which include ischemic stroke, neoplasms, Wilson’s disease, and infectious or autoimmune encephalitis [7]. However, the absence of diffusion restriction and mass effect, in combination with the patient’s clinical and metabolic profile, strongly favored DS. In our case, a 61-year-old man with poorly controlled DM presented with the classic hyperkinetic movements of the right upper and lower extremities. Neuroimaging revealed an asymmetric hyperdensity of the basal ganglia on non-contrast brain CT, with a confirmatory T1 hyperintensity on MRI involving the left striatum, consistent with DS [8]. 

Neuroimaging is essential in making the diagnosis. T1-weighted hyperintensity of the putamen and caudate nucleus is one of the hallmark findings. In our patient, CT initially suggested basal ganglia hyperintensity, while MRI confirmed the T1 hyperintensity, affirming the diagnosis of DS. Interestingly, DWI is often unremarkable in the setting of DS, with no notable diffusion restriction and normal apparent diffusion coefficient values, implying that signals were due to metabolic dysfunction [9]. Rare reports describe subtle DWI changes, potentially reflecting transient, non-chronic, cytotoxic edema or coexisting ischemic events [6,10]. Our report notably had some hyperintensities on DWI. 

The pathophysiology of DS remains incompletely understood, but several mechanisms have been proposed. One widely accepted theory suggests that severe hyperglycemia leads to a depletion of GABA, one of the primary inhibitory neurotransmitters in the basal ganglia, with an increased predisposition in non-ketotic conditions. This is proposed to occur due to the lack of alternative metabolic energy molecules available [11]. Additionally, it is proposed that hyperosmolarity leads to blood-brain barrier disruption, and astrocytic swelling causes reactive gemistocytes to damage the myelin. The decreased myelin is what is believed to be the T1 shortening signal on MRI [6]. 

Although DS is most often reported in elderly Asian women, our case demonstrates that it transcends typical demographic boundaries [12]. The patient’s HbA1c of 14% and negative ketones are consistent with the metabolic profile most frequently associated with DS. Importantly, our patient responded fully to glycemic control alone, without requiring further pharmacologic intervention with agents like neuroleptics or vesicular monoamine transporter inhibitors. Thus, adequate glycemic control is one of the keystone interventions in managing DS. 

Our case aligns with the larger series describing DS as reversible with glucose correction alone, and with no reported residual neurological sequelae [13]. It also contributes to the growing awareness that DS, albeit rare, can occur outside traditional demographic profiles. Clinicians should therefore consider DS in any patient with choreiform movements with a history of uncontrolled diabetes, since prompt recognition can lead to full recovery without lasting effects. 

## Conclusions

DS is a rare but clinically significant cause of acute hemiballismus-hemichorea in patients with uncontrolled diabetes. This case highlights the diagnostic value of neuroimaging, particularly T1-weighted MRI, and the importance of distinguishing DS from other basal ganglia pathologies, such as stroke or neoplasms. While DS is classically associated with elderly Asian women, it can occur across diverse populations, like in our patient. Timely recognition and glycemic correction are key to achieving a full recovery. Increased awareness of DS among clinicians may facilitate early diagnosis and avoid mismanagement in similar presentations.

## References

[1] Narayanan S (2012). Hyperglycemia-induced hemiballismus hemichorea: a case report and brief review of the literature. J Emerg Med.

[2] Gaillard Gaillard, F F (2025). Non-ketotic hyperglycemia hemichorea. https://radiopaedia.org/articles/non-ketotic-hyperglycaemic-hemichorea?lang=us.

[3] Shan DE, Ho DM, Chang C, Pan HC, Teng MM (1998). Hemichorea-hemiballism: an explanation for MR signal changes. AJNR Am J Neuroradiol.

[4] Park G, Kesserwani HN (2022). A case report of diabetic striatopathy: an approach to diagnosis based on clinical and radiological findings. Cureus.

[5] Chatterjee S, Ghosh R, Biswas P (2024). Diabetic striatopathy and other acute onset de novo movement disorders in hyperglycemia. Diabetes Metab Syndr.

[6] Arecco A, Ottaviani S, Boschetti M, Renzetti P, Marinelli L (2024). Diabetic striatopathy: an updated overview of current knowledge and future perspectives. J Endocrinol Invest.

[7] Rocha Cabrero F, De Jesus O (2025). Hemiballismus. StatPearls [Internet].

[8] Oh SH, Lee KY, Im JH, Lee MS (2002). Chorea associated with non-ketotic hyperglycemia and hyperintensity basal ganglia lesion on T1-weighted brain MRI study: a meta-analysis of 53 cases including four present cases. J Neurol Sci.

[9] Nahid E, Gupta S, Prasad K, Saha AK, Meher MP, Meena LP (2023). Diabetic striatopathy in an adult with ketotic hyperglycaemia. Natl Med J India.

[10] Christensen MJ, Huff TJ, Pickrell AM, Rogers SN (2025). Simultaneously occurring diabetic striatopathy and osmotic demyelination syndrome: a rare case report. Neuroradiol J.

[11] Chua CB, Sun CK, Hsu CW, Tai YC, Liang CY, Tsai IT (2020). "Diabetic striatopathy": clinical presentations, controversy, pathogenesis, treatments, and outcomes. Sci Rep.

[12] Cosentino C, Torres L, Nuñez Y, Suarez R, Velez M, Flores M (2016). Hemichorea/hemiballism associated with hyperglycemia: report of 20 cases. Tremor Other Hyperkinet Mov (N Y).

[13] Dong M, E JY, Zhang L, Teng W, Tian L (2021). Non-ketotic hyperglycemia chorea-Ballismus and intracerebral hemorrhage: a case report and literature review. Front Neurosci.

